# Lipophilic Chemicals from Diesel Exhaust Particles Trigger Calcium Response in Human Endothelial Cells via Aryl Hydrocarbon Receptor Non-Genomic Signalling

**DOI:** 10.3390/ijms19051429

**Published:** 2018-05-10

**Authors:** Bendik C. Brinchmann, Eric Le Ferrec, Normand Podechard, Dominique Lagadic-Gossmann, Kenji F. Shoji, Aubin Penna, Klara Kukowski, Alena Kubátová, Jørn A. Holme, Johan Øvrevik

**Affiliations:** 1 Department of Air Pollution and Noise, Division of Infection Control and Environmental Health, Norwegian Institute of Public Health, N-0403 Oslo, Norway; JornAndreas.Holme@fhi.no; 2 Division of Laboratory Medicine, Faculty of Medicine, University of Oslo, N-0315 Oslo, Norway; 3 Inserm, EHESP, Irset (Institut de Recherche en Santé, Environnement et Travail), Univ. Rennes, UMR_S 1085, F-35000 Rennes, France; eric.leferrec@univ-rennes1.fr (E.L.F.); norman.podechard@univ-rennes1.fr (N.P.); dominique.lagadic@univ-rennes1.fr (D.L.-G.); kenjishoji@gmail.com (K.F.S.); aubin.penna@univ-poitiers.fr (A.P.); 4 Department of Chemistry, University of North Dakota, Grand Forks, ND 58202, USA; klara.kukowski@gmail.com (K.K.); alena.kubatova@und.edu (A.K.)

**Keywords:** diesel exhaust particle extracts, endothelial cells, calcium signalling, membrane microdomains, aryl hydrocarbon receptor

## Abstract

Exposure to diesel exhaust particles (DEPs) affects endothelial function and may contribute to the development of atherosclerosis and vasomotor dysfunction. As intracellular calcium concentration [Ca^2+^]*_i_* is considered important in myoendothelial signalling, we explored the effects of extractable organic matter from DEPs (DEP-EOM) on [Ca^2+^]*_i_* and membrane microstructure in endothelial cells. DEP-EOM of increasing polarity was obtained by pressurized sequential extraction of DEPs with *n*-hexane (*n*-Hex-EOM), dichloromethane (DCM-EOM), methanol, and water. Chemical analysis revealed that the majority of organic matter was extracted by the *n*-Hex- and DCM-EOM, with polycyclic aromatic hydrocarbons primarily occurring in *n*-Hex-EOM. The concentration of calcium was measured in human microvascular endothelial cells (HMEC-1) using micro-spectrofluorometry. The lipophilic *n*-Hex-EOM and DCM-EOM, but not the more polar methanol- and water-soluble extracts, induced rapid [Ca^2+^]*_i_* increases in HMEC-1. *n*-Hex-EOM triggered [Ca^2+^]*_i_* increase from intracellular stores, followed by extracellular calcium influx consistent with store operated calcium entry (SOCE). By contrast, the less lipophilic DCM-EOM triggered [Ca^2+^]*_i_* increase via extracellular influx alone, resembling receptor operated calcium entry (ROCE). Both extracts increased [Ca^2+^]*_i_* via aryl hydrocarbon receptor (AhR) non-genomic signalling, verified by pharmacological inhibition and RNA-interference. Moreover, DCM-EOM appeared to induce an AhR-dependent reduction in the global plasma membrane order, as visualized by confocal fluorescence microscopy. DCM-EOM-triggered [Ca^2+^]*_i_* increase and membrane alterations were attenuated by the membrane stabilizing lipid cholesterol. In conclusion, lipophilic constituents of DEPs extracted by *n*-hexane and DCM seem to induce rapid AhR-dependent [Ca^2+^]*_i_* increase in HMEC-1 endothelial cells, possibly involving both ROCE and SOCE-mediated mechanisms. The semi-lipophilic fraction extracted by DCM also caused an AhR-dependent reduction in global membrane order, which appeared to be connected to the [Ca^2+^]*_i_* increase.

## 1. Introduction

Long-term exposure to air pollution, especially particulate matter (PM), is associated with progression and development of cardiovascular disease (CVD) [1]. This has largely been attributed to PM-induced oxidative stress and inflammation [2,3,4]. Human studies have found that PM exposure disturbs vascular function and systemic hemodynamics, possibly leading to hypertension [1,2,5]. PM exposure has also been linked with atherosclerosis progression, augmented vasoconstriction, and reduced vasorelaxation in arteries in vivo [6]. Humans exposed to inhalation of diesel exhaust particles (DEP) develop mild systemic inflammation and persistent endothelial dysfunction [7].

Soluble organic chemicals (OC), such as polycyclic aromatic hydrocarbons (PAHs), appear to be central to the biological effects from DEPs and other combustion-derived PM, including vascular outcomes [8,9,10,11]. Levels of urinary PAH biomarkers have been associated with CVD in US adults [12], and a link between exposure to PAHs and pre-hypertension has been reported in children [13]. Furthermore, benzo[*a*]pyrene (B[*a*]P), often used as an indicator of PAHs [14], was associated with blood pressure elevation in coke oven workers [15]. In line with studies indicating PAHs adhered to inhaled particles may diffuse into the bloodstream [14,16,17], recent findings from our laboratory suggest that lipophilic DEP-OC may penetrate the epithelial layer of a 3D tri-culture mimicking the alveolar barrier and affect endothelial cells, triggering pro-inflammatory responses [18]. 

The aryl hydrocarbon receptor (AhR) is a basic helix-loop-helix Per-Arnt-Sim (PAS) transcription factor activated by a number of chemicals found in DEPs [19]. The AhR is important in vascular function and development, and AhR-activation has been linked to disrupted endothelial function and CVD [20,21]. The prototypical AhR-ligand 2,3,7,8-tetrachlorodibenzodioxin (TCDD) has also been shown to induce hypertension and vascular dysfunction [22]. In its classical mode of action, ligand-activated AhR translocates to the nucleus, dimerizes with the AhR nuclear translocator (ARNT) and binds to xenobiotic response elements (XREs) in the promotor region of target genes. The effects of AhR activation is ligand dependent, and may also trigger non-classical, ARNT-independent genomic, as well as non-genomic, signalling pathways [23,24,25,26,27]. Notably, AhR non-genomic pathways may also regulate gene expression. The term merely reflects the AhR’s ability to act as a signalling mediator and not a transcription factor. It has been argued that inflammatory effects of TCDD are initiated by a rapid increase in intracellular calcium via AhR-non-genomic signalling [26].

The cytosolic concentration of calcium ([Ca^2+^]*_i_*) in endothelial cells is an important second messenger regulating blood pressure and flow, angiogenesis, as well as other functions [28,29,30,31]. This occurs through stimulation of Ca^2+^-sensitive K^+^-channels and myoendothelial microdomains, as well as indirectly via calmodulin which activates endothelial nitric oxide synthase (eNOS) by displacing it from caveolin-1 (Cav-1) [28,32,33]. Various ligands may trigger Ca^2+^-release from endoplasmic reticulum by binding G-protein coupled receptors (GPCR) and/or receptor tyrosine kinases (RTK) further activating the inositol trisphosphate receptor (IP3R) pathway [34]. Stromal interaction molecule (STIM) senses lowering of endoplasmic Ca^2+^-levels and next activates cellular calcium entry through ion channels in the plasma membrane, often referred to as store-operated calcium entry (SOCE) [35]. SOCE is primarily mediated by entry of extracellular calcium through Orai-channels, but transient receptor potential (TRP) channels have also been suggested in this process [36,37]. The levels of [Ca^2+^]*_i_* may also be increased through activation of receptor-operated calcium entry (ROCE) without an initial depletion of internal calcium stores. In this case, receptor activation of TRP channels and other Ca^2+^ channels result in a direct influx of Ca^2+^ through the plasma membrane. 

Calcium influx through several TRP subfamilies has been considered important to the pulmonary effects of combustion-derived PM [38]. For instance, extractable organic material of DEPs increased [Ca^2+^]*_i_* in respiratory epithelial cells by activating TRP vanilloid 4 (TRPV4) channels [39]. TRP channels, including TRP ankyrin (TRPA), TRP canonical (TRPC), and TRPV, are central mediators of Ca^2+^ influx in endothelial cells, and play a prominent role in the pathophysiological processes leading to endothelial dysfunction [40,41,42,43]. Interestingly, in a human exposure study, the calcium blocker verapamil lost its vasodilatory effect after exposure to DEPs, indicating effects on [Ca^2+^]*_i_* regulation [44]. 

Recently, we reported that lipophilic extractable organic matter of DEPs (DEP-EOM) could trigger pro-inflammatory responses in human endothelial cells [18]. In the present study, we explored whether DEP-EOM of different polarity could affect [Ca^2+^]*_i_* in human microvascular endothelial cells (HMEC-1), and if so, through which mechanisms. The two extracts containing the most lipophilic organic chemicals (i.e., *n*-hexane- (*n*-Hex-EOM) and dichloromethane-EOM (DCM-EOM)) triggered rapid and substantial [Ca^2+^]*_i_* increases via AhR signalling. The initial increase in [Ca^2+^]*_i_* triggered by *n*-Hex-EOM had an intracellular origin followed by extracellular Ca^2+^ influx, whereas the [Ca^2+^]*_i_* response triggered by DCM-EOM was completely of extracellular origin and seemed related to altered plasma membrane structure. 

## 2. Results

### 2.1. Characterization of Organic Chemicals Present in DEP-EOM

**Figure 1:**
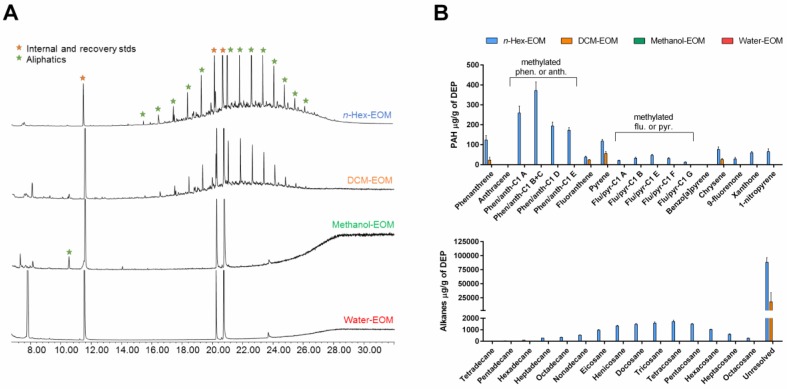
Characterization of extractable organic matter from DEPs (DEP-EOM). The soluble organic carbon/chemicals were extracted directly from the native particulates by sequential pressurized extraction with *n*-hexane, dichloromethane (DCM), and methanol at 100 °C (under pressure), followed by a final extraction with water at 25 °C. Total content of organic carbon was analyzed by thermal optical analysis, while content of polycyclic aromatic hydrocarbons (PAHs) and aliphatics were measured by gas chromatography—mass spectrometry (GC–MS), as described under Materials and Methods. The figure depicts GC-MS chromatograms of the four DEP-EOMs (**A**), as well as quantified levels of individual PAHs and alkane species of the different extracts (**B**). The extraction was done in three parallels and the results are expressed as mean ± standard error of mean (SEM) (*n* = 3).

To increase recoveries of a wide-polarity range of organic compounds from native DEPs, a sequential pressurized extraction was employed starting with non-polar *n*-hexane, followed by DCM, methanol and ending with polar water. The amount of organic carbon (OC) extracted amounted to 58 ± 6% of the total OC-content of the DEPs. Specifically, *n*-hexane recovered 27 ± 5% of OC, DCM extracted 20 ± 4%, and the methanol extract contained 11 ± 3% of OC. The OC-content of the last water extract was below detection limit. The extracted OC was dominated by aliphatic compounds, of which the majority were unresolved in the gas chromatography—mass spectrometry (GC–MS) analysis (Figure 1A). The quantified compounds included PAHs with their oxy- and nitro- derivatives, and aliphatic hydrocarbons. The majority of PAHs and their derivatives were found in the *n*-hexane extract (Table 1). The most abundant compounds included phenanthrene, methyl-phenanthrenes/anthracenes, and pyrene (Figure 1B). Besides the PAHs, *n*-hexane extract also contained the majority of recovered aliphatic hydrocarbons (i.e., saturated and unsaturated) with greater amounts of C_20_–C_26_ alkanes. Lower amounts of PAHs and aliphatic hydrocarbons were found in the DCM extract, and none were recovered from the methanol extract. Since DCM and methanol extracts contained high amounts of higher molecular weight OC (Figure S1), their identification could not be accomplished with GC–MS. Finally, besides the main constituents of DEP extracts, trace amounts of aliphatic acids were also found in the *n*-hexane, DCM, and methanol extracts. Importantly, as these extracts were obtained by sequential washing, extraction with DCM was done on particles, where *n*-hexane-soluble compounds already was removed. It should therefore be considered that if the solvents had been applied individually, or in a different order, the levels and types of compounds in each fraction would have been different, (e.g., greater OC could have been found in methanol-treated or water-treated DEPs if not previously exposed to *n*-hexane or DCM).

### 2.2. DEP-EOM Triggered Increase of [Ca^2+^]_i_ in HMEC-1

**Figure 2:**
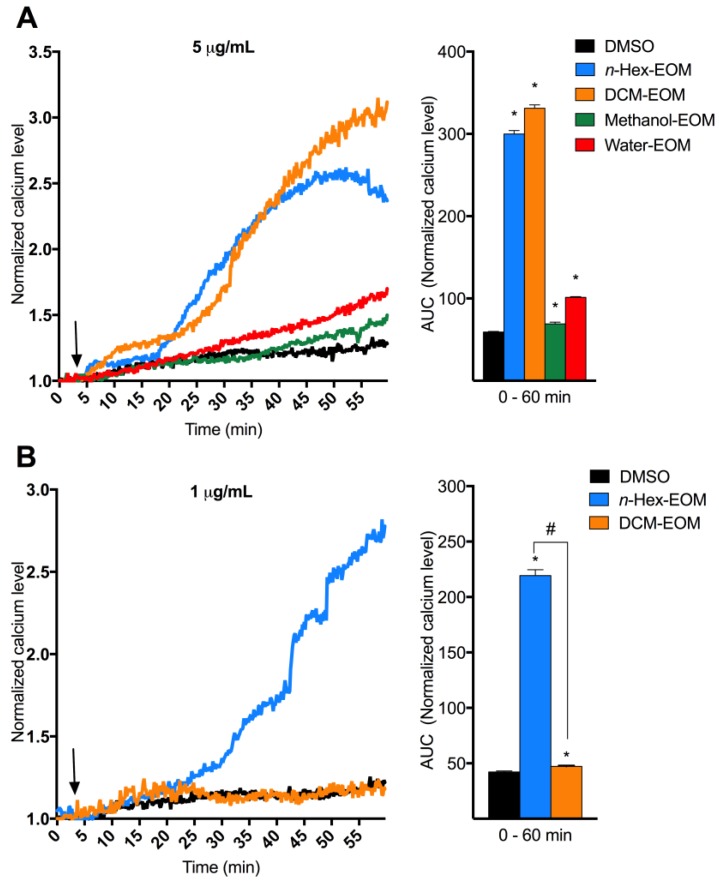
Effects of DEP-EOM on [Ca^2+^]*_i_* in human microvascular endothelial cells (HMEC-1). (**A**) Three min after measurements were started (arrow), the cells were exposed to one of the four DEP-EOM (*n*-Hex-, DCM-, Methanol or Water-EOM) at concentrations corresponding to 5 μg/mL of the original DEPs or vehicle control (DMSO); (**B**) To evaluate effects at low concentrations, three min after measurements were started (arrow), cells were exposed to *n*-Hex- and DCM-EOM at concentrations corresponding to 1 μg/mL of the original DEPs or vehicle control (DMSO). [Ca^2+^]*_i_* levels measured by normalized ratio of the Fura2-AM probe during exposure is presented as a graph and the area under the curve (AUC) from 1.0 at the *Y*-axis and 0–60 min, as mean and mean ± SEM (*n* = 3), respectively. * Significantly different from DMSO (*p* < 0.05). # Significantly different from DCM-EOM (*p* < 0.05).

Previous studies have linked both DEP and PAH exposure to [Ca^2+^]*_i_* increase [39,45,46,47]. We therefore wanted to investigate if DEP-EOM increase [Ca^2+^]*_i_* in endothelial cells. Human micro-vascular endothelial cells were exposed to four fractions of DEP-EOM ranging from non-polar to polar (*n*-Hex-EOM, DCM-EOM, Methanol-EOM, and Water-EOM). At concentrations corresponding to extracts from 5 μg/mL (0.6 μg/cm^2^) of the original DEP, *n*-Hex- and DCM-EOM triggered substantial and rapid increases of [Ca^2+^]*_i_* in HMEC-1 (Figure 2A). At a lower concentration corresponding to 1 μg/mL (0.12 μg/cm^2^) of the original DEPs, *n*-Hex-EOM still triggered substantial calcium response, while DCM-EOM had almost no effect (Figure 2B). These effects on [Ca^2+^]*_i_* were found at concentrations well below the exposure levels needed to trigger marked pro-inflammatory responses as observed in our previous study on these DEP-EOM (50 μg/mL were needed to induce IL-1 α/β, CXCL8, MMP-1 and COX-2 in HMEC-1 cells) [18].

### 2.3. Origin and Mechanisms Involved in DEP-EOM-Triggered [Ca^2+^]_i_ Increases

**Figure 3:**
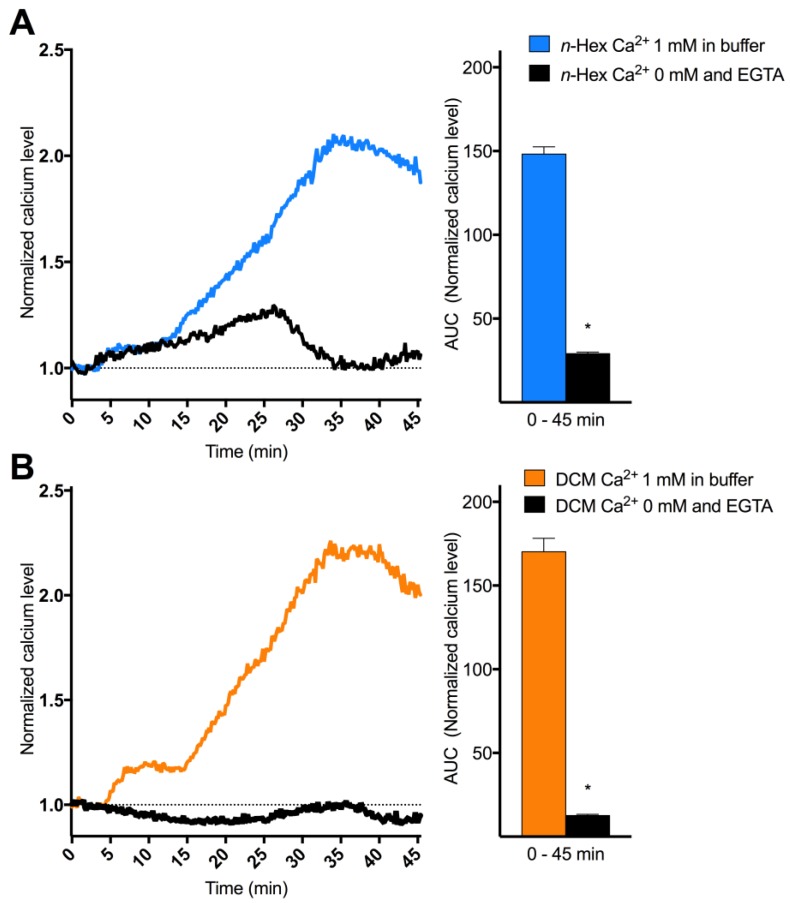
Origin and nature of *n*-Hex- and DCM-EOM-induced [Ca^2+^]*_i_* responses. Cells were incubated in a buffer with or without Ca^2+^ and ethylene glycol-bis(β-aminoethyl ether)-*N*,*N*,*N*′,*N*′-tetraacetic acid (EGTA), 30 min before exposure. Three min after measurements were started, the cells were exposed to *n*-Hex- (**A**) or DCM-EOM (**B**) at concentrations corresponding to 5 μg/mL of the original DEPs or vehicle control (DMSO). [Ca^2+^]*_i_* level measured by normalized ratio of the Fura2-AM probe during exposure is presented as a graph and AUC from 1.0 at the *Y*-axis and 0–45 min, as mean and mean ± SEM (*n* = 3), respectively. * Significantly different from calcium free exposure with EGTA (*p* < 0.05).

**Figure 4:**
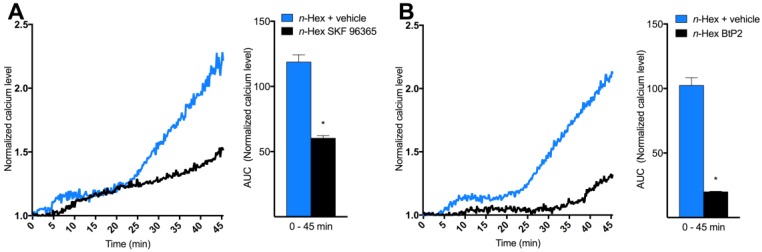

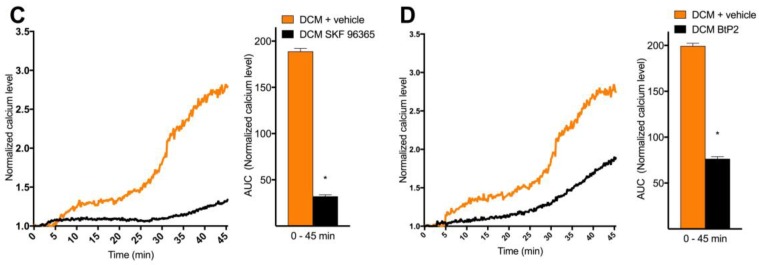
Mechanisms and channels involved in *n*-Hex- and DCM-EOM-induced [Ca^2+^]*_i_*. Cells were incubated in a buffer with or without the calcium inhibitors (i.e., 10 μM SKF 96365 or 10 μMBtP2) 30 min prior to exposure. Three min after measurements were started, the cells were exposed to *n*-Hex- (**A**,**B**) or DCM-EOM (**C**,**D**) at concentrations corresponding to 5 μg/mL of the original DEPs or vehicle control (DMSO). [Ca^2+^]*_i_* level measured by normalized ratio of the Fura2-AM probe during exposure are presented as a graph, and AUC from 1.0 at the *Y*-axis and 0–45 min, as mean and mean ± SEM (*n* = 3), respectively. * Significantly different from no inhibitor (*p* < 0.05).

To explore the origin and nature of DEP-EOM-triggered Ca^2+^ responses, HMEC-1 cells were exposed to *n*-Hex- or DCM-EOM (5 μg/mL) in buffered solution with or without Ca^2+^. The initial [Ca^2+^]*_i_* increase due to *n*-Hex-EOM was unaffected, while the latter part was abrogated by removing extracellular Ca^2+^ (Figure 3A). The STIM1/TRPC blocker SKF 96365 (10 μM) reduced the second phase of the *n*-Hex-EOM triggered effect (Figure 4A,B), consistent with inhibition of the secondary extracellular influx. Surprisingly, the SOCE/CRAC inhibitor BtP2 (10 μM) reduced both the initial as well as the latter part of this response. Ca^2+^-influx triggered by DCM-EOM was fully abrogated by removing extracellular Ca^2+^ (Figure 3B), and was almost completely blocked by SKF 96365 and partially reduced by BtP2 (Figure 4B,C). The importance of TRPC and possibly STIM1 in mediating the DCM effect was further substantiated by a marked reduction in cells treated with 50 μM of 2-APB (Figure S2) [48,49]. Thus, it seems that *n*-Hex-EOM increased [Ca^2+^]*_i_* through mechanisms consistent with SOCE while effects of DCM-EOM appeared independent of an initial Ca^2+^ release from intracellular stores, and completely due to influx of extracellular Ca^2+^, thus resembling ROCE.

**Figure 5:**
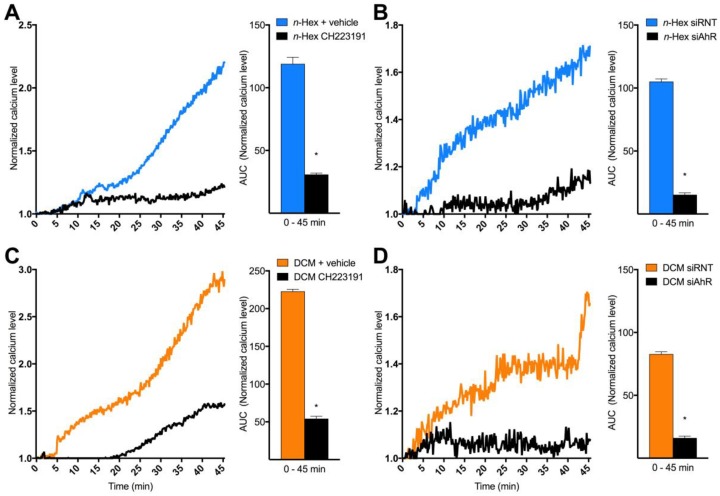
The role of AhR in [Ca^2+^]*_i_* triggered by *n*-Hex- and DCM-EOM. Cells were incubated in a buffer with or without the AhR inhibitor (1 μM CH223191; **A**,**C**) 30 min prior to exposure. AhR was knocked down with siRNA (siAhR; **B**,**D**) and control-cells treated with non-target siRNA (siRNT). Three min after measurements were started, the cells were exposed to *n*-Hex- (**A**,**B**) or DCM-EOM (**C**,**D**) at concentrations corresponding to 5 μg/mL of the original DEPs or vehicle control (DMSO). [Ca^2+^]*_i_* level measured by normalized ratio of the Fura2-AM probe during exposure are presented as a graph and AUC from 1.0 at the Y-axis and 0–45 min, as mean and mean ± SEM (*n* = 3), respectively. * Significantly different from no inhibitor (*p* < 0.05).

A rapid increase in [Ca^2+^]*_i_* via non-genomic AhR-dependent mechanisms has been suggested as an initial triggering event for TCDD-induced effects [26]. To further elucidate the mechanisms regulating [Ca^2+^]*_i_* increased by *n*-Hex- and DCM-EOM, AhR was inhibited with CH223191 (1 μM) and by siRNA knock-down. The efficiency of the siRNA-transfection has previously been verified, resulting in an approximately 85% reduction in AhR mRNA levels, loss of AhR protein expression. And a nearly complete abrogation of CYP1B1 inducibility in the HMEC-1 cells [45]. Effects of both *n*-Hex- and DCM-EOM were substantially reduced in cells treated with CH223191, but the very first part of the *n*-Hex-EOM triggered [Ca^2+^]*_i_* increase was seemingly less affected by the treatment (Figure 5A,B). However, Ca^2+^-responses to both *n*-Hex- and DCM-EOM were abrogated in cells treated with AhR specific siRNA, while cells treated with non-target siRNA still elicited a [Ca^2+^]*_i_* increase (Figure 5C,D). Thus, Ca^2+^ responses to both lipophilic DEP-EOM were dependent on AhR non-genomic signalling.

### 2.4. DCM-EOM Induced Effects on Membrane Order, Mechanisms Involved and Link to [Ca^2+^]_i_

**Figure 6:**
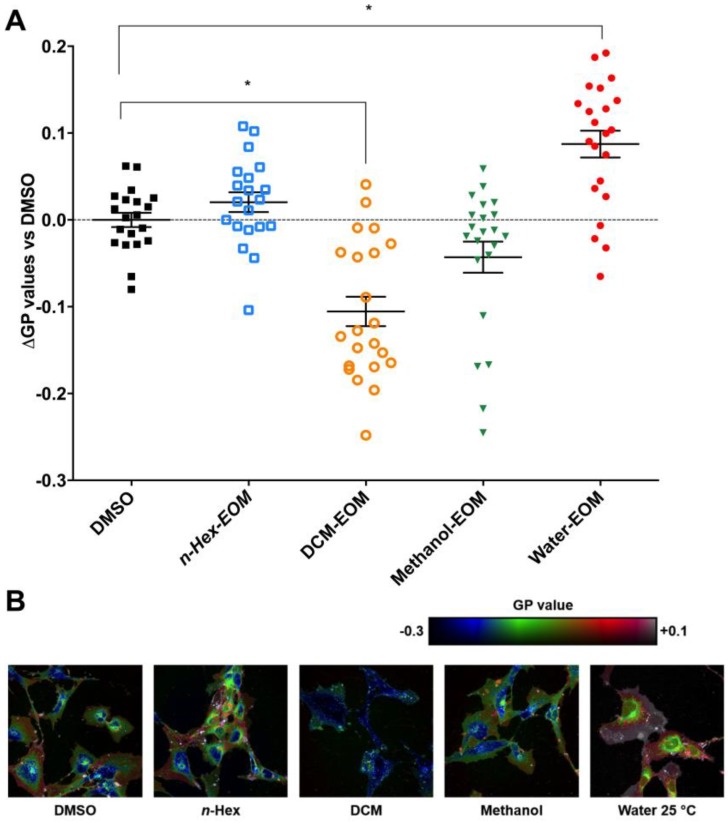
Effects of DEP-EOM on membrane-microstructure. (**A**) Cells were exposed to one of the four fractions at concentrations corresponding to 5 μg/mL of the original DEPs or vehicle control (DMSO). After 30 min exposure, cells were fixed and stained for membrane order with the fluorescence dye di-4-ANEPPDHQ; (**B**) Confocal image of HMEC-1 cells (400× magnification) stained with di-4-ANEPPDHQ, representative for three experiments. The results are expressed as mean ± SEM (*n* = 3). * Significant difference from DMSO (*p* < 0.05).

**Figure 7:**
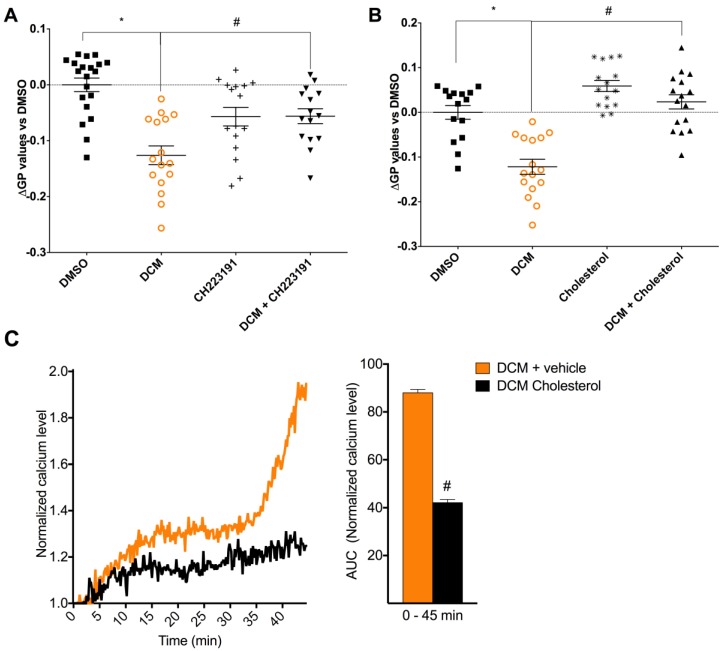
Effects of AhR inhibition and cholesterol on DCM-EOM-induced effects on membrane-microstructure and effect of cholesterol on DCM-EOM induced [Ca^2+^]*_i_*. Cells were incubated in medium with or without the AhR inhibitor (1 μM CH223191) (**A**) or cholesterol (**B**,**C**) 30 min prior to exposure. Cells were exposed to DCM-EOM (5 μg/mL) or vehicle control (DMSO). After 30 min exposure, cells were fixed and stained for lipid membrane order with the fluorescence dye di-4-ANEPPDHQ (**A**,**B**). Confocal image of HMEC-1 cells stained with di-4-ANEPPDHQ, representative for three experiments. [Ca^2+^]*_i_* levels measured by normalized ratio of the Fura2-AM probe during exposure are presented as a graph and AUC from 1.0 at the Y-axis and 0–45 min (**C**). The results are expressed as mean ± SEM (*n* = 3). * Significant difference from DMSO (*p* < 0.05). # Significant difference from DCM-EOM (*p* < 0.05).

Many key proteins regulating Ca^2+^ influx are co-localized in ordered lipid microdomains of the plasma membrane, such as caveolae [50]. We therefore also investigated the effects of DEP-EOM on plasma membrane structure. Cells exposed for 30 min to DCM-EOM (5 μg/mL) showed a reduction in di-4ANEPPDHQ-staining, indicative of a reduction in global plasma membrane order (Figure 6). Notably, Water-EOM seemed to increase membrane order, but since Water-EOM did not affect [Ca^2+^]*_i_*, the mechanism for this response was not investigated. Furthermore, pre-treatment with CH223191 (1 μM) attenuated the DCM-EOM induced membrane-remodelling (Figure 7A), indicating that this response was related to AhR signalling. It seems that the physico-chemical properties of the membrane bilayer may affect the conductance of membrane-bound channels [51]. Thus, to link our findings on membrane order to calcium, we used cholesterol (15 μg/mL), a lipid known to render the membrane more rigid [52]. As expected, addition of cholesterol prevented the DCM-EOM-induced alterations in plasma membrane order (Figure 7B). Interestingly cholesterol also inhibited [Ca^2+^]*_i_* increased by DCM-EOM, especially the latter part (Figure 7C). Thus, it seems that DCM-EOM affects membrane order, partly through AhR non-genomic signalling, and that this change in membrane order is linked to the Ca^2+^ response.

## 3. Discussion

In the current study, we found that relatively low concentrations of lipophilic DEP-EOM (*n*-Hex- and DCM-EOM) triggered a rapid increase in [Ca^2+^]*_i_* in human microvascular endothelial cells (HMEC-1) via AhR. By contrast, the more polar methanol-EOM and water-EOM did not affect [Ca^2+^]*_i_* at the tested concentrations. The relatively rapid kinetic of this response, occurring within minutes of exposure, and thus preceding gene expression, is consistent with AhR non-genomic Ca^2+^ signalling [26]. Furthermore, *n*-Hex-EOM triggered [Ca^2+^]*_i_* increase from intracellular stores followed by extracellular influx, possibly via TRP channels. In contrast, DCM-EOM-triggered [Ca^2+^]*_i_* increase appeared to be totally dependent on extracellular influx, primarily through TRP channels. The DCM-EOM-induced Ca^2+^ signalling seemed to involve an AhR-dependent reduction of ordered membrane microdomains globally. To the best of our knowledge, the present study is the first to report a potential role of AhR non-genomic signalling in the biological effects of DEPs. It is interesting to note that previous studies have found that PM-induced inflammatory and vascular effects, as well as atherosclerosis, are linked to the more volatile, lipophilic OC of the particles [11,53,54,55]. Corroborating this, our present results showed that lipophilic chemicals present in DEPs, but not the more polar constituents, increased [Ca^2+^]*_i_* in endothelial HMEC-1 cells. The apparently central role of AhR in regulating these [Ca^2+^]*_i_* increases, induced by both *n*-Hex- and DCM-EOM, further suggest that aromatic compounds may be the prime drivers of the Ca^2+^ responses by the two fractions. As seen from the chemical analysis, *n*-Hex-EOM contained approximately 10-fold higher levels of PAHs than the slightly less lipophilic DCM-EOM. Accordingly, only *n*-Hex-EOM induced a [Ca^2+^]*_i_*-increase in HMEC-1 cells when the exposure was reduced from an extract concentration corresponding to 5 μg/mL of original DEPs to 1 μg/mL.

Based on the results obtained with buffered solution containing Ca^2+^ or not, effects of *n*-Hex- and DCM-EOM on [Ca^2+^]*_i_* seem to be mediated by different mechanisms. While *n*-Hex-EOM appeared to trigger a classical IP3R-SOCE mediated response, with an initial [Ca^2+^]*_i_* increase released from the endoplasmic reticulum, DCM-EOM triggered an increase in [Ca^2+^]*_i_* that originated from the extracellular space. In line with this, the TRPC inhibitor SKF96365 had more effect on DCM-EOM-induced response than on *n*-Hex-EOM, while the SOCE inhibitor BtP2 had the most effect on *n*-Hex-EOM. The role of TRPCs in mediating the influx of extracellular Ca^2+^ triggered by DCM-EOM is further substantiated by the marked effect of 2APB (SOCE/TRPC inhibitor) (Figure S2) [32], and the presence of TRPC1 confirmed by q-PCR (Figure S3). Since BtP2 is known to inhibit SOCE/CRAC, it was somewhat surprising that it also affected the *n*-Hex-EOM triggered release of Ca^2+^ from intracellular stores. This might either be explained by a concentration related effect of BtP2, or BtP2 might inhibit release of Ca^2+^ from intracellular stores in HMEC-1. The effect of SKF 96365 on the latter part of *n*-Hex-EOM-triggered [Ca^2+^]*_i_* increase, suggests that the secondary influx of extracellular Ca^2+^ was mediated by TRPCs. This is consistent with TRPC1 acting as an integral component of SOCE [56,57]. However, SKF96365 also act as a TRPV2 and -4 antagonist [58], and has been reported to be a potent blocker of low-voltage-activated T-type calcium channels [59]. Thus the effects of SKF should be interpreted with caution. TRPV4 has both been shown to regulate DEP-induced calcium responses [39] and to play an important physiological role in endothelial cells [60]. It is therefore tempting to speculate that TRPV4 could be involved in the observed responses. However, TRPV4 was expressed at low levels in the HMEC-1 cells (Figure S3), indicating that other TRP-channels may be more important. Thus, further studies are needed to specify the role of the various TRPs. Taken together it seems that *n*-Hex-EOM triggered the classical SOCE pathway, while DCM-EOM possibly triggered a more receptor operated (ROCE) dependent pathway. Interestingly, based on their central role in vascular function, as well as in vascular pathologies, TRP channels have been suggested as potential therapeutic targets in CVD [41].

How AhR may regulate two apparently different modes of Ca^2+^ signalling by two different DEP-EOMs remains to be clarified. However, it should be noted that SOCE and ROCE share many of the same molecular mechanisms and both are activated by phospholipase C (PLC), which has been postulated as a potential downstream mediator of AhR [37,61,62]. The different mechanisms involved in the *n*-Hex- and DCM-EOM induced Ca^2+^ signaling may be linked to their chemical composition, as it is well known that various PM/PM constituents may trigger toxic responses through different mechanisms [63,64,65]. However, as *n*-Hex-EOM contains more organic chemicals than DCM-EOM, the differences in response could therefore also merely be a matter of concentration. Indeed such concentration-dependent regulation of SOCE/ROCE has been reported for other stimulants (e.g., low concentrations of endothelin, a potent vasoconstriction peptide, induced influx of extracellular Ca^2+^ only, while at higher concentrations it also released calcium from intracellular stores [66]).

An AhR non-genomic pathway has been proposed to mediate effects via both phospholipase A2/Ca^2+^ [63,64] and FAK/Src [27]. Accumulating evidence suggest that AhR localize in caveolae [33] cholesterol-rich lipid domains that are considered central in orchestrating Ca^2+^ signalling by co-localizing proteins involved [50]. Rey-Barroso and colleagues [65] have found that AhR co-localizes with caveolin-1 (Cav1) in ordered microdomains, and regulate Cav1 distribution to these microdomains. Moreover, Liao et al. [61] have suggested that Orai-TRPC complexes recruited to structured lipid domains, such as rafts or caveolae, mediate SOCE, whereas the same complexes mediate ROCE when they are outside of raft-like regions. In line with this, DCM-EOM, which appeared to induce a ROCE-like Ca^2+^ influx, also seemed to have a disordering effect on the plasma membrane, as measured by di-4ANEPPDHQ fluorescence. Notably, it has been argued that di-4ANEPPDHQ reacts specifically to cholesterol [67]. Thus, the observed effects could be indicative of caveolae disassembly and/or reduction in plasma membrane cholesterol. Pre-treatment with either cholesterol or CH223191 attenuated DCM-EOM induced reduction in global membrane order, as well as [Ca^2+^]*_i_* increase. A possible explanation is that DCM-EOM triggered an AhR-dependent disassembly of structured lipid domains, resulting in localization of TRPC-channels in non-raft regions. According to Liao et al. [61], such a scenario would favor a ROCE-mediated Ca^2+^ influx, as observed in DCM-EOM exposed cells. However, the apparent lack of effects from *n*-Hex-EOM on plasma membrane structure, despite its ability to induce AhR-dependent [Ca^2+^]*_i_*, underscores that AhR non-genomic effects may not be restricted to a single signalling pathway. 

AhR-mediated gene expression is previously reported to be among the most sensitive endpoints induced by DEPs [68,69]. Our present findings show a potential role of AhR non-genomic signalling for effects of low concentrations of DEP-EOM in endothelial cells. The significance of this relatively rapid [Ca^2+^]*_i_* increase for long-term effects of PM-exposure on endothelial function should be interpreted with caution. However, it is well known that endothelial [Ca^2+^]*_i_* plays a central role in vasomotor regulation [28,30,41]. In fact, increased SOCE-mediated Ca^2+^ influx through TRPC-channels has been found in monocytes isolated from rats and patients with hypertension [70,71]. Vasomotor dysfunction is associated with PM-exposure [6,29,72], and diesel exhaust attenuate the vasodilatory effect of the calcium blocker verapamil [44,73]. Thus, the presently observed disturbance of Ca^2+^ homeostasis may add some possible explanations to these findings. Furthermore, AhR non-genomic Ca^2+^ signalling was originally suggested by Matsumura [26] as an initial trigger of inflammatory effects upon TCDD exposure. In fact, a number of studies show that AhR-mediated induction of pro-inflammatory genes by both TCDD and PAHs depends on Ca^2+^ signalling [74,75]. Thus, the observed effects of DEP-EOM on Ca^2+^ signalling in HMEC-1 could also be related to inflammation, which plays a central role in development of endothelial dysfunction [3,76]. In line with this, we have observed that the lipophilic DEP-EOM tested in the current study also induces increased expression of pro-inflammatory genes in HMEC-1, as well as primary human endothelial cells through AhR-dependent mechanisms [16]. Recent results from our lab suggest that these pro-inflammatory responses can at least partly be attributed to the Ca^2+^ signalling, but also that enhanced calcium signalling may occur without a corresponding enhancement of the inflammatory reactions [77]. Thus, calcium signaling most likely affect regulation of pro-inflammatory genes by acting in concert with other signalling pathways. As we have previously reported that primary human endothelial cells are more sensitive to DEP-EOM than HMEC-1 with regard to inflammatory responses [16], it would thus be interesting to see if these primary cells also are more susceptible to calcium responses.

A number of studies indicate that organic chemicals are major contributors to the cardiovascular effects of DEPs and other combustion particles [4,11,18,53,78,79]. Upon inhalation and alveolar deposition of DEPs, these soluble organic chemicals may rapidly diffuse from the particles through the alveolar-endothelial barrier and into the bloodstream [14,16,17], and hence, also reach the endothelium in the more distant part of the vasculature. In vivo pulmonary exposure to B[*a*]P-coated soot particles showed that 30% of the deposited particle-bound PAH was cleared into the blood within minutes after exposure [17]. By comparison, only 0.02% of inhaled nano-sized gold particles translocated from the lung into circulation [80,81]. It therefore seems likely that the release of organic chemicals from DEPs deposited in the airways (on the epithelial surface) may represents a more important route of endothelial exposure, rather than direct exposure to DEPs (i.e., translocation of particles). Furthermore, it seems that reducing the amount of un-fully combusted organic chemicals in the air might reduce some of the health burden due to combustion derived air pollution. Elucidating what components of air pollution cause adverse health effects, and through what mechanisms this occurs, may have implications for risk assessment as well as future combustion and cleansing technology.

## 4. Material and Methods

### 4.1. Chemicals

For water extractions, deionized water was obtained from a Direct-Q purifier (Millipore, Billerica, MA, USA). All organic solvents were of >99% purity (GC or LC-MS grade) and purchased from VWR (Radnor, PA, USA). Analytical standards were obtained from either Fisher Scientific (Hampton, NH, USA) or Sigma-Aldrich (St. Louis, MO, USA). *N*,*O*-bistrifluoroacetamide (BSTFA) was also from Sigma-Aldrich (St. Louis, MO, USA).

Dimethyl sulfoxide (DMSO) and hydrocortisone were purchased from Sigma-Aldrich (St. Louis, MO, USA). l-Glutamine (200 mM) was purchased from Thermo Fischer Scientific (Renfrew, UK); endothelial growth factor were from Nerliens Meszansky (Oslo, Norway); penicillin and streptomycin were obtained from Lonza (Walkersville, MD, USA); MCDB 131 medium was provided by Life Technologies (Grand Island, NY, USA); and fetal calf serum (FCS) was from Biochrom AG (Berlin, Germany). Pluronic acid and fura-2 acetoxymethylester (Fura-2-AM) were provided by InVitrogen (Carlsbad, CA, USA). 1-[2-(4-Methoxyphenyl)-2-[3-(4-methoxyphenyl)propoxy]ethyl]-1*H*-imidazole hydrochloride (SKF 96365) and *N*-[4-[3,5-Bis(trifluoromethyl)-1*H*-pyrazol-1-yl]phenyl]-4-methyl-1,2,3-thiadiazole-5-carboxamide (BtP2) purchased from TOCRIS (Bristol, UK). 2-Aminoethoxydiphenylborate (2-APB), 2-methyl-2H-pyrazole-3-carboxylic acid (2-methyl-4-o-tolylazo-phenyl)-amide (CH223191), cholesterol, and ethylene glycol-bis(β-aminoethyl ether)-*N*,*N*,*N*′,*N*′-tetraacetic acid (EGTA) were purchased from Sigma-Aldrich (now Merck; Darmstadt, Germany). Di-4ANEPPDHQ was purchased from Molecular Probes, Life Technologies (Courtaboeuf, France).

Calcium cell suspension buffer contained: 134.8 mM NaCl, 4.7 mM KCl, 1.2 mM K_2_HPO_4_, 1 mM MgCl_2_, 1 mM CaCl_2_, 10 mM glucose, 10 mM HEPES, pH 7.4.

### 4.2. Diesel Exhaust Particles and Chemical Extraction

The DEPs were collected from the tail-pipe of a diesel engine (Deutz, 4 cylinder, 2.2 l, Cologne, Germany, 500 rpm) running on gas oil and previously chemically characterized as described elsewhere [82]. These particles, kindly provided by F.R. Cassee (RIVM, Bilthoven, The Netherlands), contained approximately 60% OC, corresponding to other OC-rich DEPs [83,84]. When compared to PM, it seems that PM_2.5_ had an OC level of approximately 25%, while ultrafine PM, known to be major contributors to vascular effects, have OC levels around 50% [85]. DEP extraction was performed from the native particles with a series of solvents ranging from non-polar to polar using *n*-hexane (*n*-Hex), dichloromethane (DCM), methanol, and water at 25 °C using a pressurized extraction system as previously described [18,86]. The solvents were removed by evaporation and the extracted DEP-EOM were re-suspended in DMSO at concentrations corresponding to extracts from 25 mg/mL of the original DEPs. The chemical compositions of these four fractions, from here on referred to as *n*-Hex-EOM, DCM-EOM, Methanol-EOM, and Water-EOM, were analyzed for total content of carbon, amount of PAHs (and their derivatives), and aliphatic hydrocarbons (Tables S1 and S2 ,and Figure S1). As shown in Table 1, most of the organic carbon was found in the *n*-Hex-EOM and DCM-EOM, with remaining 19% recovered in Methanol-EOM. Organic carbon was not detected in the Water-EOM. *N*-Hex-EOM contained almost 90% of the PAHs and aliphatic hydrocarbons, while the rest was found in the DCM-EOM [18]. 

### 4.3. Chemical Analysis

Chemical Analysis: Gas chromatography—mass spectrometry (GC–MS) revealed mainly aliphatic hydrocarbons, PAHs, and PAHs derivatives in the extracts. The corresponding extracts in organic solvents were spiked with deuterated recovery standards (i.e., naphthalene-d_8_, pyrene-d_10_, and 1-hydroxypyrene-d_9_) and concentrated to 200 μL under a gentle stream of nitrogen. Water aliquots were also spiked with recovery standards, but concentrated to 200 μL using a vacuum rotary evaporator (7–20 × 10^−3^ bar, 30 °C). Half of the concentrated sample (100 μL) was then spiked with an internal standard (i.e., fluoranthene-*d*_10_) and analyzed directly using GC–MS. To determine hydroxy-PAHs, the other half of the concentrated sample (100 μL) was evaporated to dryness under a gentle stream of nitrogen and mixed with 50 μL of sialylation agent, BSTFA. The mixture was then heated for 10 h at 70 °C, mixed with 50 μL of dichloromethane, and spiked with fluoranthene-d_10_. The GC–MS used was a 6890 Series II Plus GC coupled to a 5975C MS detector (Agilent, Santa Clara, CA, USA). Separations were carried out using a 22 m-long DB-5MS column with 0.25 mm internal diameter and 0.25 mm film thickness (J&W Scientific, Rancho Cordova, CA, USA) at a constant helium flow rate of 1.0 mL/min. Samples (1.0 μL) were injected in a split-less mode for 0.5 min at 250 °C. The temperature program started at 35 °C that was held for 2 min, followed by an increase to 140 °C with a 15 °C/min temperature gradient. The last step was an increase to 320 °C with a 10 °C/min temperature gradient, held for 10 min. The total run time was 37 min. The transfer line temperature was set to 280 °C. The MS data were acquired in the full scan mass range of 43–500 *m*/*z* using an electron ionization (70 eV). Quantifications were done using eight-point calibrations with the corresponding standard quantification ions listed in Tables S1 and S2. For compounds for which standards were not available the nearest isomeric standard was employed.

### 4.4. Cell Culture

Human micro-vascular endothelial cells (HMEC-1) were obtained from Laboratory of the Government Chemist (LGC Standards, Wesel, Germany). Cells were routinely maintained in MDCB131 medium containing epidermal growth factor (10 ng/mL), hydrocortisone (0.2 μg/mL), penicillin (50 unit/mL), and streptomycin (50 μg/mL), and were supplemented with 10% fetal calf serum (FCS) at 37 °C in an 95% humidified atmosphere with 5% CO_2_ with refreshment of medium every 2–3 day and passaged at near confluency twice per week. 

### 4.5. Small Interference RNA (SiRNA) Transfection

Chemically synthesized, double-stranded, ON-TARGETplus SMARTpool siRNA targeting mRNA of AhR, was purchased from Dharmacon (Chicago, IL, USA). We used non-targeting siRNA as a control. As previously reported [45], semi-confluent cells were transfected with 100 nM siRNA using Dharmafect-1 transfection reagent diluted in antibiotic-free culture medium, 48 h after transfection, cells were exposed to DEP-EOM.

### 4.6. Calcium Measurements

Human micro-vascular endothelial cells were grown on glass lamellas to 50–60% confluency and serum starved for a minimum of 12 h prior to exposure. Before exposure cells were mounted in exposure chambers containing 1 mL cell suspension buffer. Cells were washed two times with the buffer before loading with Fura-2AM for 30 min. Inhibitors were added during this loading period, and after the loading buffer had been washed off. The inhibitors were used at concentrations based on prior studies by others and us (2-APB 50 μM, SKF96365 10 μM, BtP2 10 μM and CH223191 1 μM) [74,87,88,89]. Calcium-free cell suspension buffer containing the extracellular calcium chelator ethylene glycol tetra acetic acid (EGTA) was used after loading in experiments on extracellular Ca^2+^ dependency.

Variations in intracellular Ca^2+^ concentrations [Ca^2+^]*_i_* were analyzed in HMEC-1 cells exposed to all four DEP-OE, by micro-spectrofluorimetry using the Ca^2+^ sensitive probe Fura-2AM, as previously reported [90]. Briefly, cells were incubated at 37 °C in cell suspension buffer supplemented with 1.5 μM Fura-2AM and 0.006% pluronic acid. After 30 min loading, cells were washed two times with the buffer before exposure. The [Ca^2+^]*_i_* imaging involved data acquisition every 10 s (emission at 510 nm) at 340- and 380-nm excitation wavelengths using an LEICA DMIRB microscope with an inverse 40× oil objective.

Changes in [Ca^2+^]*_i_* were monitored using a DMIRB (Leica, Wetzlar, Germany) inverted microscope-based imaging system equipped with a 40×/1.35 UApo N340 high UV light transmittance oil immersion objective (Olympus, Waltham, MA, USA), a CoolSnapHQ fast-cooled monochromatic digital camera (Princeton instrument), a DG-4 Ultra High Speed Wavelength Switcher (Sutter Instruments, Novato, CA, USA) for fluorophore excitation, and METAFLUOR software (Universal Imaging, Downingtown, PA, USA) for image acquisition and analysis. Data were acquired every 10 s (emission at 510 nm) at 340- and 380-nm excitation wavelengths. All images were background-subtracted. Results are presented as normalized calcium level compared to basal [Ca^2+^]*_i_* measured 3 min prior to exposure. Area under the curve (AUC) was calculated from baseline (1.0).

### 4.7. Determination of Structural Perturbation of Plasma Membrane

We visualized the plasma membrane order with confocal fluorescence microscopy using di-4 ANEPPDHQ dye (Molecular Probes, Life Technologies) a membrane-order sensitive probe. Di-4 ANEPPDHQ displays a fluorescent spectral blue-shift from 620 nm when incorporated into membranes with a low lipid order (a liquid disordered phase, Ld), and to 560 nm when inserted into membranes with a high lipid order (a liquid-ordered phase, Lo). Fluorescence images were acquired using confocal fluorescence microscopy. We acquired both disordered and ordered-phase fluorescence images, and a new image indicative of membrane order was obtained by calculating a generalized polarization (GP) value—a ratio-metric measurement of fluorescence intensities for each pixel which is correlated to membrane lipid order [91]. Briefly, after each treatment, HMEC-1 cells grown on glass lamellas were washed in phosphate buffer saline (PBS) and then fixed in 4% paraformaldehyde in PBS at 4 °C. After washing three times with PBS, the cells were stained with 5 μM di-4ANEPPDHQ for 90 min and washed twice in PBS. Cells were then visualized with confocal fluorescence microscope LEICA DMI 6000 CS (Leica Microsystems, Wetzlar, Germany). Under excitation at 488 nm with an argon ion laser, ordered membrane images were acquired with a photomultiplier tubes (PMT) range of 500 to 580 nm, whereas for disordered membrane images, the PMT range was 620 to 750 nm (magnification 400×). Using Fiji imaging processing software (ImageJ; National Institutes of Health, Bethesda, MD, USA) and the macro published by [91]), GP images were generated according to the following calculation: GP = (I_500–580_ − I_620–750_)/(I_500–580_ + I_620–750_). On each GP image generated (containing usually between one to three cells), a GP value was measured and normalized by subtraction of the mean of all GP values found for DMSO-treated cells (ΔGP values). Then, ΔGP values of images from at least three experiments were used to generate a dot blot. Finally, the global mean of ΔGP values for each condition was calculated from a minimum of 10 images.

### 4.8. Statistical Analysis

Calcium assays were analyzed by calculating area under the curve (AUC) and performing statistical comparison with unpaired *t*-test based on analysis of independent experiments (biological replicates). Due to the inherent variability in di-4-ANEPPDHQ-staining among individual cells, a very high counting-number is needed to obtain statistical significance in the membrane order measurements by confocal microscopy (Figure 6 and Figure 7). The statistical analysis in these experiments was therefore based on pooled technical replicates from three or more independent experiments. Statistical analyses were performed by GraphPad Prism 7 software (GraphPad Software, Inc., San Diego, CA, USA) using one-way ANOVA with Holm-Sidak post-test for multiple comparisons. 

## 5. Conclusions

This study demonstrated, for the first time in human microvascular endothelial cells, that exposure to lipophilic organic chemicals from diesel exhaust particles increased intracellular Ca^2+^ via AhR non-genomic signalling. Considering the variety of effects of Ca^2+^ signalling on endothelial cell physiology and pathophysiology, we believe these findings warrant further study. 

## Figures and Tables

**Table 1:** Chemical characteristics of DEP-EOMs. Total OC, aliphatic hydrocarbons (Aliphatics), and PAHs in the different DEP-EOM from [16]. Data are expressed as mean ± SD of three separate extractions. All values in mg/g of original native DEPs.

Chemicals	*n*-Hex-EOM	DCM-EOM	Methanol-EOM	Water-EOM
OC	153 ± 29	113 ± 23	62 ± 15	ND
Aliphatics	100 ± 8	17 ± 16	ND	ND
PAHs	1.65 ± 0.07	0.13 ± 0.02	ND	ND

ND = not detected; OC = organic carbon; PAHs = polycyclic aromatic hydrocarbons; DEPs = diesel exhaust particles; DCM = dichloromethane; EOM = extractable organic matter.

## Supplementary Materials

Supplementary materials can be found at http://www.mdpi.com/1422-0067/19/5/1429/s1.

## Abbreviations

AhR	aryl hydrocarbon receptor
B[*a*]P	benzo[*a*]pyrene
β2AR	β2ARadrenergic receptors
CVD	cardiovascular disease
Cav1	caveolin-1
CYP	cytochrome P450
Ca^2+^	calcium
[Ca^2+^]*_i_*	intracytoplasmic calcium concentration
OC	organic chemicals
DEPs	diesel exhaust particles
DEP-EOM	extractable organic matter of DEP
DCM-EOM	dichloromethane
DMSO	dimethyl sulfoxide
HMEC-1	human microvascular endothelial cells
IP3R	inositol trisphosphate receptor
GPCRs	G-protein coupled receptors
Methanol-EOM	methanol
NF-κB	nuclear factor-κB
*n*-Hex-EOM	DEP-EOM extracted by: *n*-hexane
PAHs	polycyclic aromatic hydrocarbons
PHECs	primary human endothelial cells
PM	particulate matter
ROS	reactive oxygen species
Water-EOM	water
XREs	AhR-response elements
