# Supplementary material for: Lipophilic Chemicals from Diesel Exhaust Particles Trigger Calcium Response in Human Endothelial Cells via Aryl Hydrocarbon Receptor Non-Genomic Signalling
Source: Int J Mol Sci. 2018 May 10;19(5):1429. doi: 10.3390/ijms19051429 (PMC5983734; doi:10.3390/ijms19051429)
Supplement: Supplementary file 1 [file ijms-19-01429-s001.pdf]

Supplementary Materials

Table S1. GC–MS quantified PAHs with corresponding MS ions and calibration standards.

Group	Compound	Formula	Quantification Ion *m/z*	Confirmation Ions *m/z*		Calibration Standard
PAHs	phenanthrene	$C_{14}H_{10}$	178	152	89	phenanthrene
	anthracene	$C_{14}H_{10}$	178	152	89	anthracene
	methyl-phenanthrene/anthracene (A–E)	$C_{15}H_{12}$	192	191	165	2-methylanthracene
	fluoranthene	$C_{16}H_{10}$	202	106	92	fluoranthene
	pyrene	$C_{16}H_{10}$	202	174	101	pyrene
	methyl-fluoranthene/pyrene (A–G)	$C_{17}H_{12}$	216	215	190	1-methylpyrene
	benzo[a]anthracene	$C_{18}H_{12}$	228	114	101	chrysene
	chrysene	$C_{18}H_{12}$	228	114	101	chrysene
	benzo[b]fluoranthene	$C_{20}H_{12}$	252	126	113	benzo[b]fluoranthene
	benzo[a]pyrene	$C_{20}H_{12}$	252	126	113	benzo[a]pyrene
Oxy-PAHs	9-fluorenone	$C_{13}H_8O$	180	152	126	9-fluorenone
	xanthone	$C_{13}H_8O_2$	196	168	139	xanthone
	anthrone (A–E)	$C_{14}H_{10}O$	194	165	139	anthrone
Hydroxy-PAHs	2-hydroxybiphenyl	$C_{12}H_{10}O$	242	227	211	2-hydroxybiphenyl
	3-hydroxybiphenyl	$C_{12}H_{10}O$	242	227	211	4-hydroxybiphenyl
	4-hydroxybiphenyl	$C_{12}H_{10}O$	242	227	211	4-hydroxybiphenyl
	2-hydroxy-9-fluorenone	$C_{13}H_8O_2$	268	195		2-hydroxy-9-fluorenone
	9-phenanthrol	$C_{14}H_{10}O$	266	251	235	9-phenanthrol
	1-hydroxypyrene	$C_{16}H_{10}O$	290	275	250	1-hydroxypyrene
Nitro-PAHs	1-nitropyrene	$C_{16}H_9NO_2$	247	231	215	1-nitropyrene

Table S2. GC-MS quantified alkanes with corresponding MS ions and calibration standards.

Alkanes	Formula	Quantification Ion *m/z*	Confirmation Ions *m/z*		Calibration Standard
tetradecane	$C_{14}H_{30}$	57	85	198	tetradecane
pentadecane	$C_{15}H_{32}$	57	85	212	tetradecane
hexadecane	$C_{16}H_{34}$	57	85	226	tetradecane
heptadecane	$C_{17}H_{36}$	57	85	240	tetradecane
octadecane	$C_{18}H_{38}$	57	85	254	tetradecane
nonadecane	$C_{19}H_{40}$	57	85	268	tetradecane
eicosane	$C_{20}H_{42}$	57	85	282	eicosane
henicosane	$C_{21}H_{44}$	57	85	296	eicosane
docosane	$C_{22}H_{46}$	57	85	310	eicosane
tricosane	$C_{23}H_{48}$	57	85	324	eicosane
tetracosane	$C_{24}H_{50}$	57	85	338	eicosane
pentacosane	$C_{25}H_{52}$	57	85	352	eicosane
hexacosane	$C_{26}H_{54}$	57	85	366	eicosane
heptacosane	$C_{27}H_{56}$	57	85	380	eicosane
octacosane	$C_{28}H_{58}$	57	85	394	eicosane

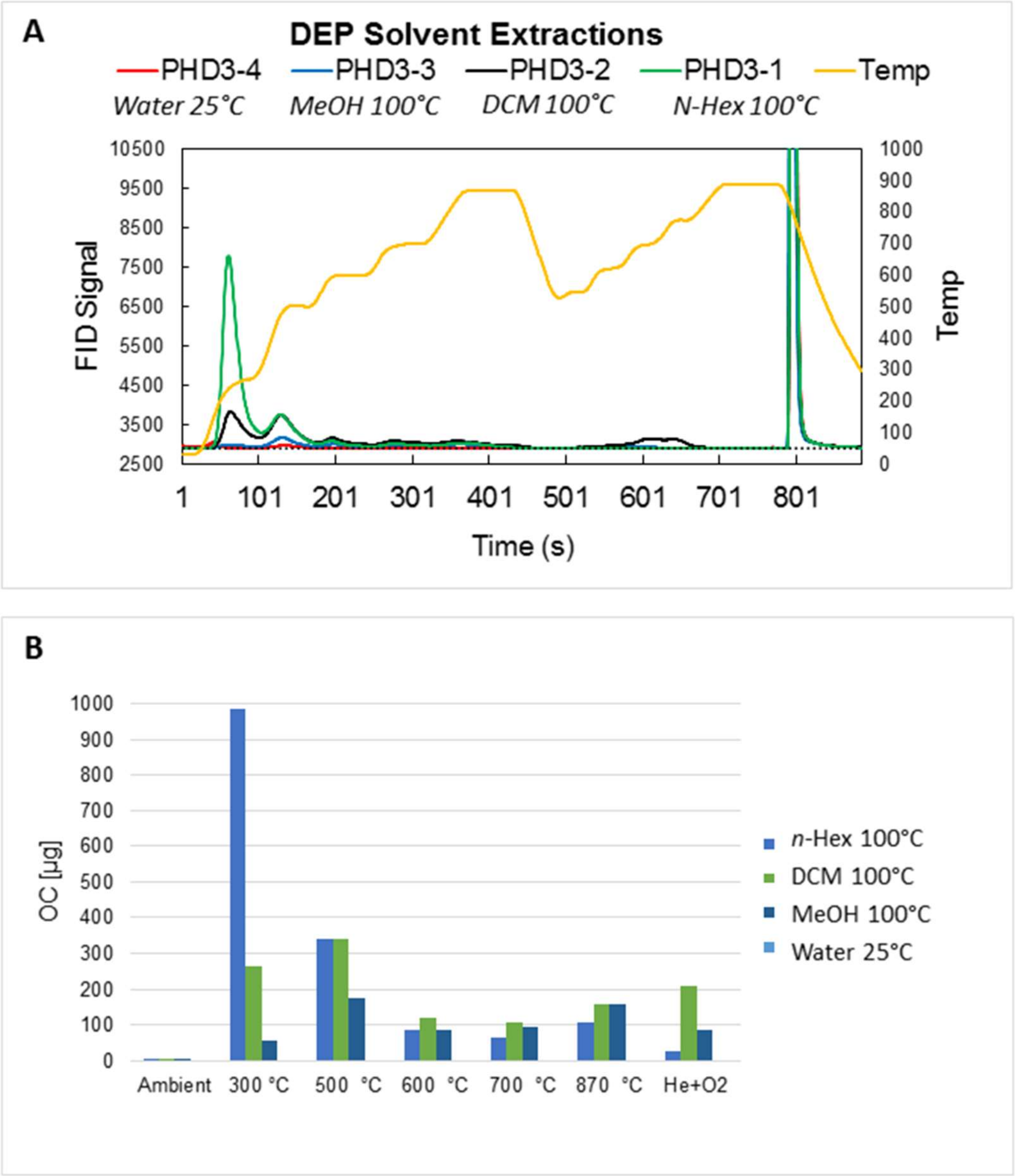

Figure S1. The amount of volatile/semi-volatile compounds extracted decreased according to polarity of the solvents. Organic carbon speciation using thermal optical analyzer (TOA) for different polarity solvent fractions obtained by DEP extraction shown as (**A**) overlaid thermograms and (**B**) processed data (ratios of peak areas converted to µg). The temperatures shown are the steps used for thermal desorption and pyrolytic evolution of carbonaceous species within TOA analysis. The results are based on EOM from 10 mg of the original DEP. The figure has previously been published elsewhere [16].

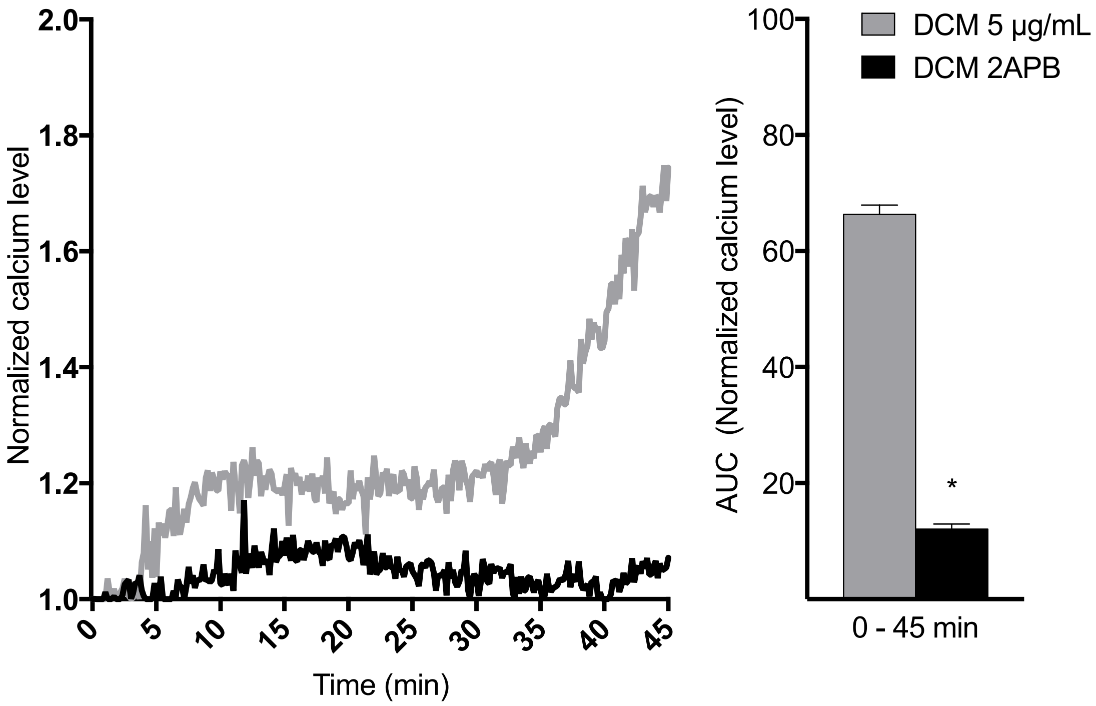

Figure S2. Effect of 2-APB on $[Ca^{2+}]_i$ triggered by DCM-EOM. Cells were incubated in buffer with or without the STIM1/TRPC inhibitor 2-APB (50 μM) 30 min prior to exposure. Three minutes after measurements were started, the cells were exposed to DCM-EOM at concentrations corresponding to 5 μg/mL of the original DEP or vehicle control (DMSO). $[Ca^{2+}]_i$ levels measured by normalized ratio of the Fura2-AM probe during exposure is presented as graph and the area under the curve (AUC) 0–45 min, as mean and mean ± SEM (n = 3). * Significantly different from no inhibitor.

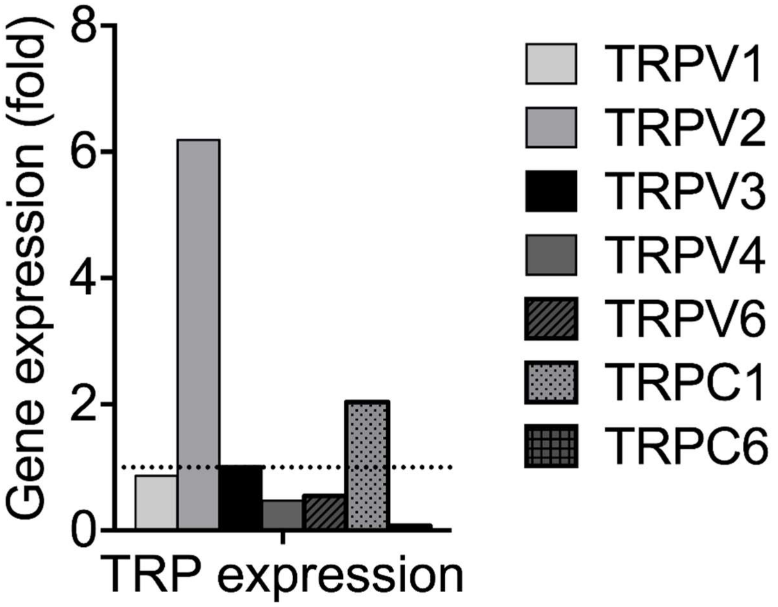

Figure S3. Basic expression of calcium conductance channels in HMEC-1. Unexposed cells were harvested, mRNA isolated and gene expression measured with qPCR (n = 1). Expression is relative to the average of all seven genes.
